# VDAC1: A Key Player in the Mitochondrial Landscape of Neurodegeneration

**DOI:** 10.3390/biom15010033

**Published:** 2024-12-30

**Authors:** Shirel Argueti-Ostrovsky, Shir Barel, Joy Kahn, Adrian Israelson

**Affiliations:** 1 Department of Physiology and Cell Biology, Faculty of Health Sciences, Ben-Gurion University of the Negev, P.O. Box 653, Beer Sheva 84105, Israel; 2 The School of Brain Sciences and Cognition, Ben-Gurion University of the Negev, P.O. Box 653, Beer Sheva 84105, Israel

**Keywords:** VDAC1, neurodegenerative diseases, ALS, Parkinson’s disease, Alzheimer’s disease

## Abstract

Voltage-Dependent Anion Channel 1 (VDAC1) is a mitochondrial outer membrane protein that plays a crucial role in regulating cellular energy metabolism and apoptosis by mediating the exchange of ions and metabolites between mitochondria and the cytosol. Mitochondrial dysfunction and oxidative stress are central features of neurodegenerative diseases. The pivotal functions of VDAC1 in controlling mitochondrial membrane permeability, regulating calcium balance, and facilitating programmed cell death pathways, position it as a key determinant in the delicate balance between neuronal viability and degeneration. Accordingly, increasing evidence suggests that VDAC1 is implicated in the pathophysiology of neurodegenerative diseases, including Alzheimer’s disease (AD), Parkinson’s disease (PD), amyotrophic lateral sclerosis (ALS), and others. This review summarizes the current findings on the contribution of VDAC1 to neurodegeneration, focusing on its interactions with disease-specific proteins, such as amyloid-β, α-synuclein, and mutant SOD1. By unraveling the complex involvement of VDAC1 in neurodegenerative processes, this review highlights potential avenues for future research and drug development aimed at alleviating mitochondrial-related neurodegeneration.

## 1. The Role of VDAC1 in Mitochondrial Function, and Its Relevance to Neurodegenerative Diseases

Mitochondria are vital organelles that play a primary role in ATP production, thus powering cellular functions [1]. Additionally, they play a crucial role in cellular signaling, cell division, and apoptosis [2]. The complex architecture of the mitochondrion is essential for its proper and efficient function. Mitochondria are characterized by two distinct membranes: the outer mitochondrial membrane (OMM) and the inner mitochondrial membrane (IMM), which are divided by the intermembrane space (IMS) [3]. The central line for the transit of metabolites and nucleotides across the OMM is the voltage-dependent anion channel (VDAC), also referred to as mitochondrial porin.

Three isoforms of VDAC in mammals—VDAC1, VDAC2, and VDAC3—have been identified, all of which share several functional and structural characteristics. VDACs, as β-barrel proteins composed of antiparallel amphipathic β-strands, form hydrophilic pores in the mitochondrial outer membrane [4]. These isoforms create selective, voltage-dependent ion channels that mediate the transport of nucleotides (ATP, ADP, AMP, NADH), ionic metabolites (pyruvate, glutamate, succinate, malate), and lipids, and allow for the passage of inorganic ions (K^+^, Na^+^, Ca^2+^, Cl^−^) and certain organic ions. Beyond their role in regulating the exchange of ions and metabolites between mitochondria and the cytosol, VDACs also influence a wide array of intracellular processes [5,6,7]. VDAC1 is a key mediator of mitochondria-driven apoptosis, while VDAC2, conversely, serves an anti-apoptotic function. It is likely that these two proteins, by co-localizing within specific subcompartments of the OMM, jointly regulate the balance between pro-apoptotic and anti-apoptotic signals in the cell. Although the role of VDAC3 in apoptosis initiation remains unclear, this isoform has been implicated in the regulation of reactive oxygen species (ROS) production and mitochondrial quality control [8].

Mitochondrial dysfunction is widely recognized as a significant contributor to various neurodegenerative diseases, including Alzheimer’s disease (AD), Parkinson’s disease (PD), amyotrophic lateral sclerosis (ALS), Huntington’s disease (HD), etc. [9]. A primary mechanism by which mitochondria contribute to neurodegeneration is through the excessive production of ROS, leading to oxidative stress—a common pathological feature underlying these disorders [10]. Elevated ROS levels can also directly damage mitochondrial components, particularly mitochondrial DNA (mtDNA) [11], further exacerbating the neurodegenerative process. While mitochondria are primarily known for their role in energy production and cellular metabolism, they also serve as crucial regulators of apoptosis [12], which is crucial for maintaining cellular homeostasis. Dysregulation of apoptosis, whether through excessive or insufficient activation, has been implicated in various neuropathological conditions [13,14].

At the intersection of these critical mitochondrial functions lies the VDAC1 channel, the most abundant and extensively studied isoform. This protein has emerged as a key mediator in the complex interplay between mitochondrial function, oxidative stress, and apoptosis regulation in the context of neurodegenerative diseases [4] and, thus, will be the focus of this review.

**Figure 1:**
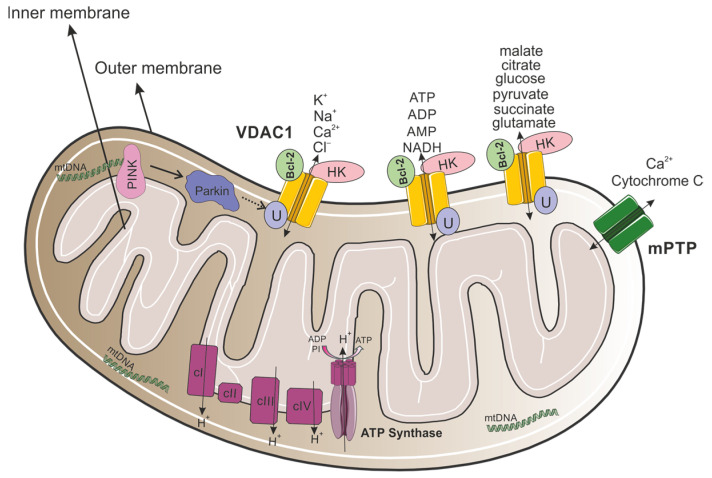
VDAC1 as a key modulator of mitochondrial metabolism, apoptosis, and quality control. The mitochondrion contains an outer and inner membrane, with an intermembrane space between them. The outer membrane includes VDAC1 channels that allow for the passage of ions (K^+^, Na^+^, Ca^2+^, Cl^−^) and metabolites (such as ATP, ADP, AMP, NADH, malate, citrate, glucose, pyruvate, succinate, and glutamate). Bcl-2 and HK are associated with VDAC1, regulating cellular apoptosis and glucose metabolism. Proteins such as PINK and Parkin are involved in mitochondrial quality control, specifically in mitophagy and apoptosis. VDAC1 serves as a substrate for Parkin-mediated ubiquitination where monoubiquitination inhibits apoptosis, while polyubiquitination promotes mitophagy. The mitochondrial permeability transition pore (mPTP) facilitates the release of Ca^2+^ and cytochrome c, which are critical for initiating apoptosis. Within the inner membrane, complexes I-IV of the electron transport chain generate a proton gradient (H^+^) across the membrane. ATP synthase uses this gradient to synthesize ATP from ADP and inorganic phosphate (Pi). Mitochondrial DNA (mtDNA) is also shown, illustrating its role in encoding essential proteins for mitochondrial function.

VDAC1 is a multifunctional channel that is located in the OMM (Figure 1), regulating the metabolic and energetic crosstalk between mitochondria and the rest of the cell, which plays a role in mitochondria-mediated apoptosis [15]. VDAC1, which possesses Ca^2+^ and Ruthenium Red binding sites [16,17], is also involved in cholesterol transportation, regulating lipid metabolism, mediating ion channels, regulating Ca^2+^ signaling between mitochondria and the endoplasmic reticulum (ER), and regulating the redox status of mitochondria and the cytoplasm [18].

The atomic-resolution structure of VDAC1 reveals a β-barrel composed of 19 transmembrane β-strands, with strands β1 and β19 in parallel conformation. The 25-residue N-terminal region resides inside the pore and is proposed to translocate from the internal pore to the channel surface, modulating the conductance of ions and metabolites [19,20]. The pore diameter is estimated between 3 and 3.8 nm, narrowing to approximately 1.5 nm when the N-terminal α-helix occupies the pore. A glycine-rich motif (21GlyTyrGlyPheGly25) connects the N-terminal domain to β-strand 1, providing the flexibility necessary for N-terminal translocation [21]. This N-terminal mobility is implicated in channel gating, VDAC1 dimerization, interactions with anti-apoptotic proteins, and binding to apoptosis-regulating proteins from the Bcl-2 family (e.g., Bax, Bcl-2, Bcl-xL) and hexokinase [22].

This review aims to elucidate the specific roles of VDAC1 in the pathogenesis of AD, PD, ALS, and HD, exploring its potential as both a therapeutic target and a biomarker for these disorders.

## 2. VDAC1 in Alzheimer’s Disease

Alzheimer’s disease (AD), the most prevalent neurodegenerative disease and most common form of dementia, is characterized by a gradual, age-related deterioration of brain function that leads to impaired memory, cognitive decline, and alterations in behavior [23,24]. This disease represents a growing global health challenge, with 50 million cases reported in 2018 and projected to triple by 2050 [25]. The pathological hallmarks of AD include extracellular amyloid plaques composed primarily of aggregated amyloid-beta (Aβ) peptides, intracellular neurofibrillary tangles consisting of hyperphosphorylated tau protein, and significant neuronal loss [26]. While genetic mutations in *amyloid precursor protein* (*APP*), *presenilin 1* (*PS1*) and *presenilin 2* (*PS2*) genes cause less than 2% of all AD cases, the majority are late-onset sporadic cases, suggesting a complex interplay between genetic and environmental factors [27,28]. AD pathogenesis involves multiple molecular and cellular events, including synaptic dysfunction, synaptic loss [29,30], and mitochondrial abnormalities [31,32].

Mitochondrial dysfunction is considered an early event in AD pathogenesis, with several physiological events noted in AD brains, such as changes in mitochondrial DNA (mtDNA), decreased mitochondrial enzyme activity, abnormal mitochondrial gene expression, increased mitochondrial fragmentation, decreased mitochondrial fusion, reduced metabolism, disruption of Ca^2+^ homeostasis, increased production of ROS, lipid peroxidation, and apoptosis [29,33,34,35,36,37,38,39]. Compelling evidence for the critical role of mitochondria in AD pathology has been obtained by applying cytoplasmic hybrid (‘cybrid’) technology. In these experiments, researchers created hybrid cells by replacing the endogenous mitochondria of healthy cells with mitochondria derived from AD patients. The resulting cybrid cells manifested numerous pathological hallmarks typical of AD [40].

**Figure 2:**
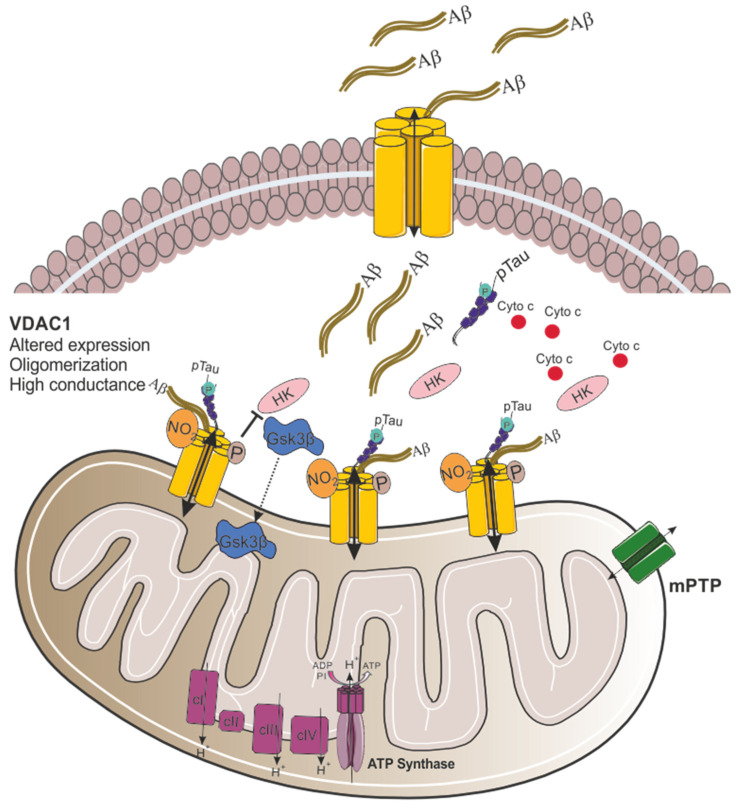
Proposed model for VDAC1 involvement in mitochondrial dysfunction in Alzheimer’s disease. In AD, the expression of VDAC1 is altered, and the channel conductance is increased. Aβ oligomers interact with VDAC1 at the plasma membrane through its N-terminal domain, leading to the formation of a VDAC1-Aβ heterooligomer that facilitates Aβ entry into the cell. Once in the cytosol, Aβ interacts with mitochondrial VDAC1, causing VDAC1 oligomerization [45]. Moreover, VDAC1 directly interacts with phosphorylated tau (pTau), further contributing to mitochondrial dysfunction. Finally, increased activity of GSK3β promotes its translocation to the mitochondria, and the phosphorylation of VDAC1 at threonine 51 (Thr51). This phosphorylation causes the detachment of HK from VDAC1, triggering cytochrome c release and promoting apoptotic cell death.

Recent research has implicated VDAC1 as the key mitochondrial gatekeeper in AD pathogenesis. Quantitative real-time-PCR and immunoblotting analyses of postmortem AD brains and APP transgenic mice revealed significantly increased VDAC1 mRNA and protein levels, correlating with disease progression and age [41]. Mechanistic insights from in vitro studies showed that Aβ peptides and oligomers induce mitochondrial damage and VDAC1 upregulation (Figure 2) [42]. Notably, AβPP transgenic mice exhibited increased levels of phosphorylated VDAC1 in hippocampal extracts [43], while AD brains showed elevated nitrated VDAC1, indicating oxidative damage (Figure 2) [44].

In addition to the elevated VDAC1 levels observed in AD cases, studies have demonstrated that VDAC1 directly interacts with Aβ and phosphorylated tau, impairing the opening and closing of mitochondrial pores, ultimately leading to mitochondrial dysfunction [41]. Immunoprecipitation analysis using cortical protein lysates from AD postmortem brains and transgenic APP and APP/PS1 mice revealed a 4 kDa Aβ and 100 kDa full-length APP in VDAC1 immunoprecipitants [41]. Moreover, monomeric and oligomeric Aβ from cortical protein lysates were detected in association with VDAC1 in severe AD patients and older APP/APP/PS1 mice [24,41]. Smilansky and colleagues demonstrated a direct interaction between Aβ and VDAC1 by surface plasmon resonance and microscale thermophoresis. By assessing VDAC1 channel conductance using a planar lipid bilayer system, it was shown that Aβ caused a twofold increase in channel conductance (Figure 2). Additionally, it was found that a VDAC1 N-terminal peptide (VDAC1-N-Ter) inhibited Aβ cell penetration and prevented Aβ-induced cell death [45]. The interaction between Aβ and VDAC1 is likely driven by electrostatic forces, with negative charges on Aβ, positive charges on the VDAC1 N-terminal domain, and hydrophobic interactions. Aβ contains three GXXXG motifs, while VDAC1 has one at its N-terminus. These motifs are thought to facilitate the interaction between the N-terminus of VDAC1 and the C-terminal region of Aβ in AD neurons. Interestingly, VDAC1-interacting proteins like Bcl-xL and Bcl-2 also possess GXXXG motifs, suggesting a more comprehensive role for this motif in protein–protein interactions [46]. Finally, a model was proposed for Aβ-induced, mitochondria-mediated cell death. In this model, plasma membrane-localized VDAC1 (pl-VDAC1) interacts with Aβ oligomers via its N-terminal domain, forming a pl-VDAC1-Aβ hetero-oligomer that facilitates Aβ entry into the cell (Figure 2). Once in the cytosol, Aβ binds to the N-terminal domain of mitochondrial VDAC1, promoting its oligomerization and, thus, leading to cytochrome c release and apoptotic cell death (Figure 2) [14].

The interaction between abnormal, phosphorylated tau and VDAC1 was also shown by immunoprecipitation using cortical protein lysates from AD patients and transgenic APP/PS1, 3xTg.AD mice. VDAC1 was detected in phosphorylated tau immunoprecipitation elutes and phosphorylated tau was observed in VDAC1 immunoprecipitants from the brains of AD patients and transgenic mice. These results, confirmed by immunofluorescence analysis, suggest a direct interaction between phosphorylated tau and VDAC1 [24,41].

As mentioned before, VDAC1 contributes to apoptosis in AD. One example of this is the increased activity of glycogen synthase kinase 3 (GSK3β) that was observed in AD resulting in increased Aβ production, and hyperphosphorylation of tau (Figure 2) [47]. GSK3β phosphorylates VDAC1, resulting in the detachment of hexokinase II (HKII) from VDAC1 and disrupting cellular metabolism (Figure 2) [48]. When HK is associated with VDAC1 in the OMM, it has direct access to ATP produced by the mitochondria, which provides protection against apoptosis [49,50]. However, once HKII detaches from VDAC1, it loses access to mitochondrial ATP, thereby decreasing the ATP available for glycolysis and glucose metabolism, which increases the cells’ susceptibility to apoptosis. Moreover, Aβ has been shown to induce HK detachment, initiating VDAC1 oligomerization, cytochrome c release, and subsequent apoptosis [14].

To inhibit VDAC1 oligomerization, pro-apoptotic protein release, ROS production, and increased intracellular Ca^2+^, Shoshan-Barmatz and colleagues developed two VDAC1-interacting compounds, VBIT-4 and VBIT-12 [51]. These compounds have been shown to prevent mitochondrial dysfunction and apoptosis in various mouse models with VDAC1 overexpressed, including type 2 diabetes [52], lupus [53], and colitis (Table 1) [54]. Additionally, it was found that VBIT-4 mitigated AD-related pathophysiological changes, such as neuronal loss, neuroinflammation, and metabolic dysfunction. Targeting overexpressed VDAC1 in AD prevents its oligomerization at an early stage of apoptosis and ameliorates all tested AD-associated pathways [55].

Moreover, a recent study suggests that reducing VDAC1 expression may alleviate mitochondrial dysfunction in AD. In transgenic tau mice, partial reduction of VDAC1 decreased mitochondrial fission proteins and increased fusion proteins, along with elevated mitochondrial protein biogenesis. These findings indicate that reducing VDAC1 expression protects against tau-induced mitochondrial toxicity, offering potential therapeutic insights for AD [65].

In summary, research has established VDAC1 as a key player in AD pathogenesis. Its increased expression correlates with disease progression and aging, while its interactions with Aβ and phosphorylated tau contribute to mitochondrial dysfunction and neuronal death. The role of VDAC1 in apoptosis and energy metabolism further underscores its importance in AD pathology. Recent studies targeting VDAC1 have shown promise in mitigating AD-related symptoms in various models, highlighting its potential as both a biomarker and a therapeutic target. Future research should focus on developing targeted interventions to modulate VDAC1 function, potentially opening new avenues for AD treatment and prevention.

## 3. VDAC1 in Parkinson’s Disease

Parkinson’s disease (PD) is a prevalent neurodegenerative disorder that leads to significant disability and contributes to a growing global public health challenge, affecting motor, non-motor, and cognitive functions [66]. PD impacts around 2–3% of individuals over 65 years of age, with the number of cases increased from 2.5 million in 1990 to 6.2 million in 2015, and is expected to reach 12.9 million cases by 2040 [67]. About 25% of individuals with PD have a disease onset before 65, and 5–10% before 50. The term “young onset” refers to cases in which the disease starts before age 40 and possibly extends to those under 50 [68]. Men are 1.5 times more likely to have PD than women [69]. Most PD cases are sporadic, with only about 10% of patients indicating a family history [70]. Among the six genes associated with heritable, monogenic PD, mutations in the genes *SNCA* (*PARK1*) and *LRRK2* (*PARK8*) are linked to autosomal-dominant forms, while mutations in *Parkin* (*PARK2*), *PINK1* (*PARK6*), *DJ-1* (*PARK7*), and *ATP13A2* (*PARK9*) are associated with autosomal recessive inheritance [71]. Moreover, mutations in the *glucocerebrosidase* (*GBA*) gene, which codes for a lysosomal enzyme implicated in Gaucher’s disease, have emerged as a prominent risk factor for PD [72]. However, even the broadest estimates suggest that the narrow-sense heritability of PD is approximately 27% [73], indicating that the risk of PD is still primarily affected by environmental factors. The hallmark neuropathological feature of PD is the progressive loss and degeneration of dopamine-producing neurons within the substantia nigra (SN) [69]. These dying dopaminergic neurons contain Lewy bodies and Lewy neurites, which represent the final stage of a cellular process, beginning with small α-synuclein aggregates observed in the neuron’s cytoplasm that then merge into diffused pale bodies [74]. This merging leads to the formation of an aggregation seed, causing diffused α-synuclein to aggregate into filaments that trap organelles such as mitochondria and lysosomes [75,76,77]. Pathogenic mutations in α-synuclein accelerate the formation of these aggregates, suggesting that α-synuclein dysfunction is an early event in both familial and likely sporadic PD [78].

Both genetic and environmental factors are implicated in mitochondrial dysfunction in the pathogenesis of PD [79]. Epidemiological studies consistently demonstrate an association between pesticide exposure and increased risk of PD, alongside reports of drug-induced parkinsonism linked to methyl-4-phenyl-1,2,3,6-tetrahydropyridine (MPTP) toxicity, indicating that specific toxins contribute to nigral damage through inhibition of mitochondrial complex I. Furthermore, the application of neurotoxins to inhibit mitochondrial complex I in animal models has yielded mechanistic evidence supporting the link between mitochondrial dysfunction and PD [79,80]. Complex I defects are a major cause of apoptosis in neurons and are also thought to be one of the main causes of PD [81].

**Figure 3:**
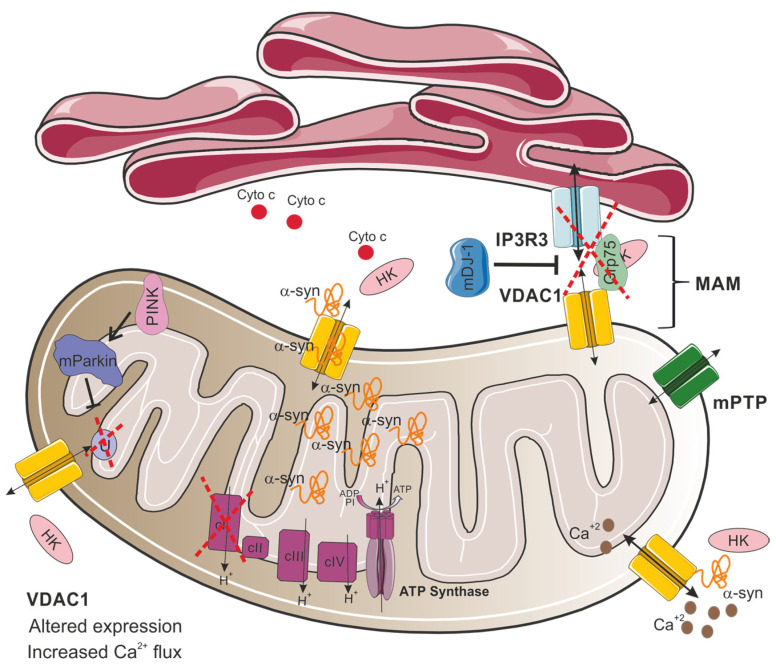
Proposed model for VDAC1 involvement in mitochondrial dysfunction in Parkinson’s disease. In PD, VDAC1 altered expression and its interactions with α-synuclein (α-syn) disrupt mitochondrial function. This interaction enables α-synuclein translocation and accumulation to the mitochondria, modulating VDAC1 permeability and increasing selective calcium flux through the VDAC1 channel, leading to mitochondrial calcium overload, dysfunction, and subsequent cell death. Additionally, VDAC1 is involved in the IP3R3-Grp75-VDAC1 complex at mitochondria-associated membranes (MAM), which mediates calcium transfer from the endoplasmic reticulum (ER) to mitochondria. This complex is destabilized by the loss of DJ-1, impairing ER–mitochondria calcium homeostasis. Mutations in Parkin inhibit VDAC1 monoubiquitination, leading to increased mitochondrial calcium influx and apoptosis, reflecting PD pathology. HK detachment from VDAC1 results in cytochrome c release, amplifying apoptotic signaling. In addition, defects in complex I of the electron transport chain are a major cause of mitochondrial malfunction in PD.

The relationship between mitochondrial dysfunction and PD also involves VDAC1, which plays a role in regulating apoptosis and autophagy, as well as interacting with the hallmark protein of the disease, α-synuclein [82]. Importantly, VDAC1 was found to be a critical component for the PINK1–Parkin pathway [83]. In this process, PINK1 facilitates Parkin recruitment to the mitochondria, initiating mitochondrial autophagy [84]. Parkin, functioning as an E3 ubiquitin ligase, mediates the degradation of misfolded proteins [85]. A study by Ham and colleagues revealed that VDAC1 is a critical substrate of Parkin. According to their research, Parkin can induce two distinct ubiquitination patterns on VDAC1: monoubiquitination, which suppresses apoptotic pathways, and polyubiquitination, which facilitates mitophagy [83]. Notably, the Parkin T415N mutation, observed in PD patients, fails to induce VDAC1 monoubiquitination in HEK-293T and mouse embryonic fibroblasts (Figure 3) [83]. Crucially, VDAC1 monoubiquitination deficiency enhances calcium influx via the mitochondrial calcium uniporter (MCU), consequently triggering apoptotic pathways [83]. Additionally, flies expressing a Parkin mutant T433N (analogous to the human T415N mutation) failed to prevent apoptosis, mirroring the cell culture findings. These flies exhibited decreased locomotor activity, altered mitochondrial morphology, and degeneration of dopaminergic neurons, similar to the symptoms observed in PD [83]. Moreover, transgenic flies expressing a VDAC1 mutant deficient in monoubiquitination (corresponding to human VDAC1K274R) displayed PD-like symptoms, including impaired movement, loss of dopaminergic neurons, and increased muscle apoptosis [83]. Thus, this study emphasizes the critical role of closely regulating VDAC1-mediated apoptosis in the pathogenesis of PD. In addition, VDAC1, by acting as a ROS sensor, modulates apoptosis through the regulation of the mitochondrial permeability transition pore (mPTP), a complex located in the inner and outer mitochondrial membrane whose opening is crucial for initiating cell death pathways [86].

In addition, VDAC1 serves as a crucial constituent of the IP3R3-Grp75-VDAC1 complex, which localizes to mitochondria-associated membranes (MAM) [87]. These specialized subcellular domains represent critical crosstalk points between the ER and mitochondria [88]. Notably, depletion of DJ-1 results in the destabilization of this macromolecular complex, consequently impairing ER–mitochondria communication (Figure 3). This disruption leads to compromised mitochondrial calcium homeostasis, specifically manifesting as reduced calcium uptake into the mitochondrial matrix, ultimately culminating in mitochondrial dysfunction [87].

In neurons, α-synuclein has been shown to bind all three VDAC isoforms [82] with the highest affinity to VDAC1 [89]. Also, single-channel electrophysiology experiments show that monomeric α-synuclein can transiently block and translocate through all VDAC isoforms [82]. Previous studies have demonstrated a direct interaction between VDAC1 and α-synuclein, suggesting that this interaction enables α-synuclein to translocate into the mitochondria (Figure 3) [90]. Furthermore, overexpression of α-synuclein in the SN of rats can lead to the degeneration of dopaminergic neurons by triggering an interaction between α-synuclein and VDAC1, causing changes in the mPTP, resulting in cell death [91]. It has been demonstrated that α-synuclein binding modulates VDAC1 permeability by increasing its selectivity for calcium. This results in an enhanced flux of Ca^2+^ through the channel, leading to mitochondrial dysfunction and cell death (Figure 3) [92].

Interestingly, while alterations in VDAC1 protein levels are well documented in various neurodegenerative disorders [43], findings specific to PD remain inconsistent. For example, Chu and colleagues found that VDAC1 expression was significantly reduced in nigral neurons of PD patients and in rat models overexpressing the mutant α-synuclein A30P [93]. Supporting these findings, a different study reported a marked decrease in VDAC1 and VDAC2 levels in SNpc samples from PD patients and in SH-SY5Y neuroblastoma cells treated with dopamine [94]. In contrast, treatment with rotenone or 1-methyl-4-phenylpyridinium (MPP+), increased VDAC1 and VDAC2 protein levels by approximately 50% in SH-SY5Y cells [94,95]. Additionally, elevated VDAC1 expression has been observed in the striatum and cortex of Parkin knockout mice [96], as well as in extracts from 6-hydroxydopamine lesions (Figure 3) [97].

Given the pivotal function of VDAC1 in regulating metabolic exchange across the OMM and its critical role in the cell apoptotic cascade, this protein represents a promising therapeutic target for various pathological conditions, including PD. For instance, olesoxime, a compound that interacts with the VDAC1 β-barrel at the lipid-protein interface, hinders the translocation of α-synuclein through the VDAC1 pore, thereby preventing mitochondrial dysfunction and the production of ROS associated with α-synuclein accumulation in mitochondria (Table 1) [58]. In addition, 4-Phenylbutyrate (4-PBA) [60] was examined as a possible therapeutic agent in a rotenone-induced PD rat model. Notably, 4-PBA prevented VDAC1 upregulation and subsequent cytochrome c release, thereby preserving mitochondrial function and preventing neuronal apoptosis (Table 1) [61]. This molecule significantly ameliorated motor deficits, reduced α-synuclein accumulation, and restored dopamine levels in the nigrostriatal pathway. Similarly, resveratrol, a potent antioxidant and neuroprotective agent, was found by Feng and colleagues to have neuroprotective effects in α-synuclein A53T transgenic mice through VDAC1 regulation. In their study, resveratrol treatment improved motor and cognitive function while reducing VDAC1 expression and its interaction with α-synuclein (Table 1). This reduction in VDAC1-α-synuclein co-localization prevented mitochondrial dysfunction by decreasing mPTP opening, oxidative stress, and α-synuclein aggregation [98]. Finally, these findings implicate VDAC1 as a promising therapeutic target for PD via modulation of mitochondrial pathways.

## 4. VDAC1 in Amyotrophic Lateral Sclerosis

Amyotrophic lateral sclerosis (ALS) is a fatal late-onset neurodegenerative disease, characterized by the loss of upper and lower motor neurons (MNs) in the brain and spinal cord. MN degeneration causes progressive denervation of skeletal muscles, resulting in muscle weakness, atrophy, and paralysis, which eventually leads to death from respiratory failure within 2–5 years after disease onset [99,100]. Most ALS cases, roughly 90%, are classified as sporadic (sALS), with no apparent family history of the disease, while the remaining 10% are categorized as familial cases (fALS) [101], attributed to mutations in more than 40 different genes [102]; among them are *superoxide dismutase 1* (*SOD1*), *chromosome 9 open reading frame 72* (*C9orf72*), and *TAR DNA-binding protein 43* (*TDP-43*) [99,102]. Multiple cellular deficits are implicated in both sporadic and familial ALS, including impaired proteostasis and protein aggregation [103,104,105], increased oxidative stress [106,107], excitotoxicity [108], and neuroinflammation [109,110,111].

Altered mitochondrial function [99,112,113,114,115], structural abnormalities of the mitochondria [115,116,117], and disruption of mitochondrial axonal transport [115,118], have also been reported as ALS-associated mechanisms. These manifest predominantly as energy production impairments, disruption of calcium homeostasis, and activation of apoptosis [99,119]. Recent studies, utilizing fibroblasts from ALS patients and iPSC-derived MNs, have reported impairments in the mitochondrial respiratory chain function and ATP production [120], as well as elevated levels of oxidative stress [121]. Furthermore, aberrant proteins involved in ALS pathogenesis were shown to interact with the mitochondria, causing mitochondrial dysfunction [117,121,122,123].

**Figure 4:**
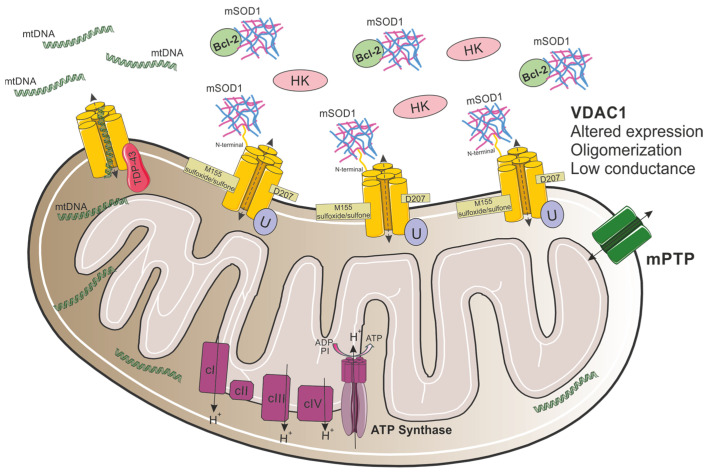
Proposed model for VDAC1 involvement in mitochondrial dysfunction in ALS. In ALS, mutant SOD1 interacts with VDAC1 through its N-terminal domain, leading to the detachment of HK1 from the channel. In addition, the association between mutant SOD1 and Bcl-2 disrupts the association between Bcl-2 and VDAC1. Moreover, VDAC1 oligomerization levels are elevated, channel conductance is reduced, and VDAC1 undergoes specific Asn^207^ deamidation and Met^155^ oxidation. In TDP-43-related ALS, TDP-43 mislocalizes into the mitochondria and interacts with VDAC1, resulting in aberrant mtDNA release to the cytosol.

Both dismutase active and inactive SOD1 mutants were found to selectively interact with VDAC1 in the spinal cord, but not the brain or liver mitochondria of symptomatic SOD1^G93A^ or SOD1^H46R^ rats (Figure 4) [123]. This interaction, through the N-terminal domain of VDAC1 [62] with 28–61 residues in SOD1 [56], reduces VDAC1 channel conductance, as demonstrated using reconstituted VDAC1 into a planar lipid bilayer [63,123], and suppresses ADP transport across the OMM, suggesting a reduction in energy supply to the spinal MNs [123]. Importantly, reducing VDAC1 activity by targeted gene disruption was shown to accelerate disease onset and diminish the survival of mutant SOD1^G37R^ mice [123]. Moreover, VDAC1 is the receptor for Hexokinase I (HKI) enzyme, which is important for energy production [124], and its interaction with VDAC1 has a protective effect against apoptotic cell death [125,126,127]. Mutant SOD1^G93A^ competes with HKI for its VDAC1 binding site, suggesting that the SOD1-VDAC1 association docks mitochondria and promotes its dysfunction (Figure 4) [63]. Decreased flux of metabolites through VDAC1 and the competition between SOD1 and HKI, likely reduce energy production and regulate apoptotic intrinsic pathways, leading to mitochondrial dysfunction [63,123].

To better understand mutant SOD1–VDAC1 interaction, post-translational modifications in VDAC1 were recently analyzed by using high-resolution mass spectrometry [128]. VDAC1 was shown to undergo specific oxidations and deamidations (Figure 4), exclusively in NSC-34-SOD1^G93A^ cells, changing the channel’s structure. These alternations affect VDAC1 physiological function, which, in turn, promotes mitochondrial dysfunction and, presumably, tags mitochondria with deaminated VDAC1 for mitophagy [128].

Nevertheless, it was proposed that VDAC1 is a downstream target for the association between mutant SOD1 and Bcl-2 [129], an ordinarily pro-survival mitochondrial protein that interacts with VDAC1. Co-immunoprecipitation experiments [129,130] in HEK293T cells, which do not express endogenous Bcl-2 [131], revealed that as Bcl-2 expression increases, the amount of mutant SOD1 pulled down with VDAC1 decreases, suggesting that SOD1 and VDAC1 compete for Bcl-2 binding [129]. In addition, knocking down VDAC1 with siRNAs did not affect SOD1/Bcl-2 interaction in those cells. Another interesting finding was that in cells expressing SOD1^G93A^ and VDAC1, but not Bcl-2, VDAC1 conductance was not significantly reduced, whereas co-expression of SOD1^G93A^ and Bcl-2 reduced channel conductance by about 40% [129]. Moreover, VDAC1-Bcl-2 binding increased as the disease progressed in SOD1^G93A^ mice due to disease-driven conformational changes in Bcl-2 structure, induced by mutant SOD1/Bcl-2 association [129,131]. Taken together, these results imply that mutant SOD1 targets VDAC1 through aberrant interaction with Bcl-2 (Figure 4) and that mutant SOD1 requires Bcl-2 to influence VDAC1 gating properties [129].

Understating mutant SOD1–VDAC1 interaction paves the way to new avenues for the development of novel therapeutic strategies. Indeed, synthetic cell-penetrating VDAC1-N-terminal-derived peptides were tested for their potential to prevent mutant SOD1-mediated toxicity in vitro [62]. Specifically, (10-20)N-Ter-Antp peptide significantly reduced apoptosis and increased cell viability of SOD1G93A or SOD1G37R expressing NSC-34 cells. Moreover, in SH-SY5Y neuronal-like cells, the co-localization of misfolded SOD1 with VDAC1 was extensively reduced after (10-20)N-Ter-Antp incubation. Likewise, SOD1^G93A^-expressing mESC-derived MNs incubated with (10-20)N-Ter-Antp peptide presented significantly increased MN neurite outgrow, improved MN density, and extended survival (Table 1) [62].

Another two VDAC1-specific small molecules, VBIT-4 and VBIT-12, were recently tested as therapeutic targets for ALS, both in vitro and in vivo (Table 1) [56]. The two molecules were previously reported to prevent VDAC1 oligomerization, a process resulting in the release of pro-apoptotic proteins, leading to cell death [51]. In fact, VDAC1 oligomeric levels were elevated in mitochondria isolated from SOD1^G93A^ mice and rats’ spinal cord [56]. Incubating mitochondria isolated from SOD1^G93A^ mice with VBIT-4 inhibited the oligomerization of VDAC1. Additionally, in SOD1^G93A^-expressing NSC-34 cells, VBIT-12 incubation partially prevented mutant SOD1 cell toxicity. However, treating SOD1^G93A^ mice with VBIT-4 or VBIT-12 administered to their drinking water had no effect on disease onset or survival, even though some improvement in muscle endurance was observed in mice treated with VBIT-12. Although intraperitoneal (IP) administration of VBIT-12 showed a positive, but not significant, tendency in delaying disease onset and survival of SOD1^G93A^ mice, the treated mice retained their limb muscle strength for a significantly extended period of time [56].

Although VDAC1 levels were found to be significantly higher in pre-symptomatic and symptomatic SOD1^G93A^ mice, specifically in spinal MNs [56], upregulation of VDAC1 was recently tested as a therapeutic approach for mutant SOD1 mice [132]. VDAC1 overexpression by AAV2/5 vector, directly injected into the spinal cord of neonatal pups, restored mitochondrial respiratory profile, probably by affecting the function of complex I enzymes, mitochondrial sirtuins, and the receptor subunit of the translocase of the outer membrane (TOM) complex, Tom20 [132]. The influence of VDAC1 upregulation on disease onset, progression, and mice survival was not indicated.

NHK1, a synthetic peptide corresponding to the first 11 amino acids of human HK1, was also proposed as a tool to impair the formation of VDAC1-SOD1^G93A^ complexes [63,64]. NHK1 peptide interacts with VDAC1 [63,64] and restores mitochondrial dysfunction by modulating channel conductance [63] and improving the respiratory profile of the mitochondria, specifically by increasing the ATP-linked oxygen flows [64] and partially recovering mitochondrial potential variation [63]. The ability of NHK1 to impair VDAC1-SOD1^G93A^ interaction reduces the accumulation of toxic SOD1^G93A^ mitochondrial aggregates [63,64], increases VDAC1 protein levels, and recovers cell viability (Table 1) [64].

Furthermore, the cholesterol-like compound, olesoxime (TRO19622), which interacts with VDAC showed positive outcomes when tested both in vitro and in vivo [59]. Olesoxime presented a neuroprotective effect on the survival of rat embryonic MNs and it accelerated the regenerative processes followed by lesions of the sciatic nerve in adult mice. This neuroprotective effect might be related to its ability to slow down neuromuscular junction (NMJ) denervation, as well as astrogliosis and microgliosis activation (Table 1) [133]. Based on the above findings, olesoxime treatment was further tested in SOD1^G93A^ mice. Olesoxime administration successfully delayed the onset of body weight decline and motor function and significantly increased mice’s life span by 10% [59]; however, it failed to significantly improve the symptoms or survival of ALS patients [134].

Notably, VDAC1 was recently linked to other forms of fALS. First, Yu and colleagues used iPSC-derived MNs from patients carrying TDP-43 mutations and showed that inhibiting VDAC1 oligomerization with VBIT-4 treatment prevents leakage of mtDNA into the cytosol [57]. Moreover, in mouse embryonic fibroblasts (MEFs) overexpressing TDP-43, VDAC1 knockout prevented the expression of Ifnb1 and Tnf, innate immune-related factors [57]. In HEK293T cells, TDP-43 was found to interact with several mitochondrial proteins, including VDAC1 [135]. These observations suggest that in ALS pathogenesis, TDP-43 can mislocalize into the mitochondria, interact with VDAC1, and cause cytosolic accumulation of mtDNA (Figure 4) [57,135]. Furthermore, VDAC1 is known to be involved in the crosstalk between the mitochondria and ER. Specifically, glucose-related protein 75 (GRP75) mediates the interaction between VDAC1 and IP3R, directly promoting mitochondria Ca^2+^ uptake [136]. In a recent study that investigated early ER stress-induced adaptive mechanisms, 2-week-old iPSC-derived MNs from C9orf72-ALS/FTD patients had elevated GRP75 transcripts, accompanied by elevated VDAC1 transcripts and increased IP3R-VDAC1 interaction [137]. Interestingly, in spinal cord of C9-500 mice, IP3R-VDAC1 association was increased at P125, followed by a decrease at P240, coinciding with GRP75 expression levels. Sustaining high GRP75 expression in C9-500 mice by AAV6-GRP75 injection prevented ER stress, normalized mitochondrial function, and enhanced IP3R-VDAC1 interaction [137]. Supporting these results, another study has recently reported a nonsignificant elevation of VDAC1 levels in skin fibroblasts of C9orf72-ALS/FTD patients; however, this was connected to increased oxidative stress and mitochondrial dysfunction [138]. Taken together, these observations strengthen the notion that neurons in C9orf72-ALS/FTD are susceptible to ER–mitochondrial dysfunction and that GRP75 serves as a critical endogenous neuroprotective factor by mediating IP3R-VDAC1 interaction [137].

In summary, the involvement of VDAC1 in ALS-related pathogenesis was described in several types of fALS, but it is mainly mediated through its interaction with mutant SOD1. This interaction impairs VDAC1 association with other proteins, alters channel function(s), and, consequently, prompts mitochondrial dysfunction. Elevated expression of VDAC1 in pre-symptomatic and symptomatic SOD1^G93A^ mice highlights the importance of VDAC1 in disease development. Importantly, several approaches were suggested to interfere with aberrant VDAC1-SOD1 interaction, thereby restoring mitochondrial function and improving cellular deficits. Testing those candidates targeting VDAC1 in vivo may serve as a potential therapeutic strategy in ALS.

## 5. VDAC1 in Other Neurodegenerative Conditions

VDAC1 has also been associated with the pathological mechanisms involved in other neurodegenerative diseases, such as Huntington’s disease (HD), a neurodegenerative disorder caused by repeat expansions in the *huntingtin* (*HTT*) gene [139,140]. In HD, the formation of mutant HTT protein leads to neuronal loss in the striatum, cortex, and hypothalamus, resulting in involuntary movements, cognitive decline, and psychiatric disturbances [139,140]. VDAC1 channel conductance was found to be reduced in mutant HTT-expressing PC12 cells [141]. In addition, in a rat model of HD, 3-NP disrupts mitochondrial dynamics and ER–mitochondria crosstalk in the striatum, increases VDAC1 expression [142], and inhibits VDAC1 conductance, thereby altering mitochondrial Ca^2+^ influx [143].

As previously discussed regarding ALS, olesoxime which binds VDAC [59,144], was also tested as a therapeutic strategy for spinal muscular atrophy (SMA), a motor neuron disease in which SMN protein deficiency results in NMJ degeneration and loss of spinal MNs [144]. Following the subcutaneous injection of olesoxime, which significantly increased lifespan in a relevant mouse model, a Phase 2 trial has confirmed human safety and tolerance [145]. Unfortunately, a long-term follow-up revealed olesoxime was ineffective in slowing functional decline [146], and the development of olesoxime as a treatment for SMA has been suspended.

Recently, an age-dependent decrease in VDAC1 protein expression in aged mice brains was reported, corresponding to hippocampal neurodegeneration and, subsequently, recognition memory impairments during aging [147]. In Batten disease, the most common autosomal-recessive neurodegenerative childhood disease, VDAC1 showed protein expression changes in the thalamus of early-symptomatic mice, which correlated with the initiation of neuronal pathology, suggesting that VDAC1 can be used as an early biomarker of modified axonal and synaptic vulnerability [148].

## 6. Conclusions

Finally, VDAC1 plays a pivotal role in mitochondrial dysfunction, as observed across various neurodegenerative diseases, including AD, PD, ALS, and others. By modulating mitochondrial permeability and participating in the regulation of apoptosis, VDAC1 significantly impacts neuronal survival under disease conditions. The findings reviewed here emphasize that targeting VDAC1 interactions, such as those with amyloid-beta, α-synuclein, mutant SOD1, and TDP-43, can potentially restore mitochondrial health and cellular energy balance. Moreover, altered VDAC1 expression, increased oligomerization, and dysregulated calcium flux are common among these diseases, leading to mitochondrial stress, ROS production, and cell death.

Several VDAC1-targeting compounds, such as VBIT-4, VBIT-12, resveratrol, and olesoxime, have demonstrated neuroprotective effects by restoring mitochondrial function, reducing ROS production, and inhibiting pathogenic protein interactions in preclinical models of AD, PD, and ALS. These therapeutic strategies highlight VDAC1’s potential to ameliorate mitochondrial dysfunction and neurodegeneration.

Future therapeutic strategies targeting VDAC1 could offer promising solutions for mitigating mitochondrial dysfunction, thus slowing down the progression of neurodegeneration and paving the way for more effective treatments. Integrating small-molecule inhibitors, peptide-based therapies, and genetic tool strategies will further expand therapeutic options. Moreover, applying advanced drug delivery systems, such as nanoparticles, could enhance the bioavailability and efficacy of VDAC1-targeted therapies. Continued research focusing on the interplay between VDAC1 and disease-specific proteins, ER–mitochondria crosstalk, and mitochondrial quality control pathways will be essential in uncovering novel mechanisms and refining therapeutic approaches. Together, these progressions hold significant potential to revolutionize the treatment of neurodegenerative diseases, offering hope for improved patient outcomes and disease management.

## Figures and Tables

**Table 1:** Potential therapeutic agents for neurodegenerative diseases targeting mitochondrial function via VDAC1.

Disease	Potential Therapeutic Agent	Agent Structure	Agent Description and Mechanism of Action
AD, ALS	VBIT-4	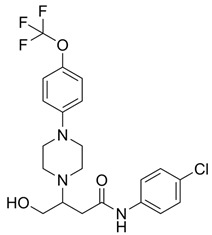	Interacts with VDAC1 and strongly inhibits its oligomerization, thereby preventing apoptosis. This action reduces ROS production, cellular Ca^2+^ levels, and the inflammatory response while also restoring cell metabolism [55,56]. In addition, VBIT-4 prevents mtDNA release to the cytosol [57].
ALS	VBIT-12	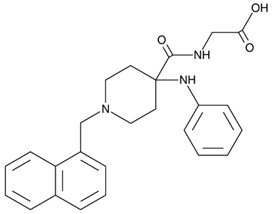	Interacts with VDAC1 and strongly inhibits its oligomerization, thereby preventing apoptosis. This action reduces ROS production, cellular Ca^2+^ levels, and the inflammatory response while also restoring cell metabolism [55,56].
PD, ALS	Olesoxime	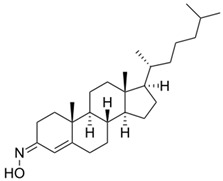	Low molecular weight, cholesterol-like compound [5]. It interacts with the VDAC1 β-barrel at the lipid-protein interface [58], has a neuroprotective effect on MN survival, and accelerates regeneration of damaged nerves [59].
PD	4-PBA	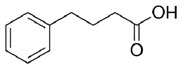	Short-chain hydrophobic fatty acid having chemical chaperone activity [60]. Inhibits VDAC1 upregulation and the subsequent release of cytochrome c, thereby maintaining mitochondrial function and preventing neuronal apoptosis [61].
PD	Resveratrol	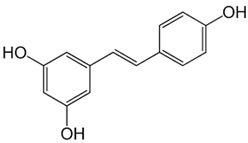	Potent phytoalexin with antioxidant and anti-inflammatory properties [11]. Reduces VDAC1 and α-synuclein expression preventing their interaction. This inhibition reduces α-synuclein accumulation within mitochondria, mitigating mitochondrial dysfunction and protecting against neuronal apoptosis [61].
ALS	(10-20)N-Ter-Antp peptide	LGKSARDVFTK	VDAC1-N-terminal-derived peptide [13]. Binds mutant SOD1 and prevents its association with VDAC1, thus inhibiting its adverse effect on channels’ function and, subsequently, reducing mitochondrial dysfunction [62].
ALS	NHK1	IAAQLLAYYFT	A synthetic peptide corresponding to the first 11 amino acids of human HK1 [14]. Impairs the interaction between VDAC1 and SOD1^G93A^, improving mitochondrial function and cell viability [63,64].

## Data Availability

Not applicable.

## References

[1] Spinelli J.B., Haigis M.C. (2018). The multifaceted contributions of mitochondria to cellular metabolism. Nat. Cell Biol..

[2] Chen W., Zhao H., Li Y. (2023). Mitochondrial dynamics in health and disease: Mechanisms and potential targets. Signal Transduct. Target. Ther..

[3] Jenkins B.C., Neikirk K., Katti P., Claypool S.M., Kirabo A., McReynolds M.R., Hinton A. (2024). Mitochondria in disease: Changes in shapes and dynamics. Trends Biochem. Sci..

[4] Shoshan-Barmatz V., Shteinfer-Kuzmine A., Verma A. (2020). VDAC1 at the Intersection of Cell Metabolism, Apoptosis, and Diseases. Biomolecules.

[5] Hodge T., Colombini M. (1997). Regulation of metabolite flux through voltage-gating of VDAC channels. J. Membr. Biol..

[6] Rostovtseva T., Colombini M. (1997). VDAC channels mediate and gate the flow of ATP: Implications for the regulation of mitochondrial function. Biophys. J..

[7] Magri A., Reina S., De Pinto V. (2018). VDAC1 as Pharmacological Target in Cancer and Neurodegeneration: Focus on Its Role in Apoptosis. Front. Chem..

[8] Belosludtseva N.V., Dubinin M.V., Belosludtsev K.N. (2024). Pore-Forming VDAC Proteins of the Outer Mitochondrial Membrane: Regulation and Pathophysiological Role. Biochemistry.

[9] Lin M.T., Beal M.F. (2006). Mitochondrial dysfunction and oxidative stress in neurodegenerative diseases. Nature.

[10] Singh A., Kukreti R., Saso L., Kukreti S. (2019). Oxidative Stress: A Key Modulator in Neurodegenerative Diseases. Molecules.

[11] Barnham K.J., Masters C.L., Bush A.I. (2004). Neurodegenerative diseases and oxidative stress. Nat. Rev. Drug Discov..

[12] Wang X. (2001). The expanding role of mitochondria in apoptosis. Genes Dev..

[13] Tatton W.G., Olanow C.W. (1999). Apoptosis in neurodegenerative diseases: The role of mitochondria. Biochim. Biophys. Acta.

[14] Shoshan-Barmatz V., Nahon-Crystal E., Shteinfer-Kuzmine A., Gupta R. (2018). VDAC1, mitochondrial dysfunction, and Alzheimer’s disease. Pharmacol. Res..

[15] Shoshan-Barmatz V., Krelin Y., Chen Q. (2017). VDAC1 as a Player in Mitochondria-Mediated Apoptosis and Target for Modulating Apoptosis. Curr. Med. Chem..

[16] Israelson A., Abu-Hamad S., Zaid H., Nahon E., Shoshan-Barmatz V. (2007). Localization of the voltage-dependent anion channel-1 Ca^2+^-binding sites. Cell Calcium.

[17] Israelson A., Zaid H., Abu-Hamad S., Nahon E., Shoshan-Barmatz V. (2008). Mapping the ruthenium red-binding site of the voltage-dependent anion channel-1. Cell Calcium.

[18] Hu H., Guo L., Overholser J., Wang X. (2022). Mitochondrial VDAC1: A Potential Therapeutic Target of Inflammation-Related Diseases and Clinical Opportunities. Cells.

[19] Bayrhuber M., Meins T., Habeck M., Becker S., Giller K., Villinger S., Vonrhein C., Griesinger C., Zweckstetter M., Zeth K. (2008). Structure of the human voltage-dependent anion channel. Proc. Natl. Acad. Sci. USA.

[20] Ujwal R., Cascio D., Colletier J.P., Faham S., Zhang J., Toro L., Ping P., Abramson J. (2008). The crystal structure of mouse VDAC1 at 2.3 A resolution reveals mechanistic insights into metabolite gating. Proc. Natl. Acad. Sci. USA.

[21] Geula S., Ben-Hail D., Shoshan-Barmatz V. (2012). Structure-based analysis of VDAC1: N-terminus location, translocation, channel gating and association with anti-apoptotic proteins. Biochem. J..

[22] Shoshan-Barmatz V., Maldonado E.N., Krelin Y. (2017). VDAC1 at the crossroads of cell metabolism, apoptosis and cell stress. Cell Stress.

[23] Lei P., Ayton S., Bush A.I. (2021). The essential elements of Alzheimer’s disease. J. Biol. Chem..

[24] Reddy P.H. (2013). Is the mitochondrial outermembrane protein VDAC1 therapeutic target for Alzheimer’s disease?. Biochim. Biophys. Acta.

[25] Scheltens P., De Strooper B., Kivipelto M., Holstege H., Chetelat G., Teunissen C.E., Cummings J., van der Flier W.M. (2021). Alzheimer’s disease. Lancet.

[26] Murphy M.P., LeVine H. (2010). Alzheimer’s disease and the amyloid-beta peptide. J. Alzheimer’s Dis..

[27] Mawuenyega K.G., Sigurdson W., Ovod V., Munsell L., Kasten T., Morris J.C., Yarasheski K.E., Bateman R.J. (2010). Decreased clearance of CNS beta-amyloid in Alzheimer’s disease. Science.

[28] Wildsmith K.R., Holley M., Savage J.C., Skerrett R., Landreth G.E. (2013). Evidence for impaired amyloid beta clearance in Alzheimer’s disease. Alzheimer’s Res. Ther..

[29] Reddy P.H., Tripathi R., Troung Q., Tirumala K., Reddy T.P., Anekonda V., Shirendeb U.P., Calkins M.J., Reddy A.P., Mao P. (2012). Abnormal mitochondrial dynamics and synaptic degeneration as early events in Alzheimer’s disease: Implications to mitochondria-targeted antioxidant therapeutics. Biochim. Biophys. Acta.

[30] Selkoe D.J. (2002). Alzheimer’s disease is a synaptic failure. Science.

[31] Swerdlow R.H. (2011). Brain aging, Alzheimer’s disease, and mitochondria. Biochim. Biophys. Acta.

[32] Zhu X., Perry G., Smith M.A., Wang X. (2013). Abnormal mitochondrial dynamics in the pathogenesis of Alzheimer’s disease. J. Alzheimer’s Dis..

[33] Quintanilla R.A., Dolan P.J., Jin Y.N., Johnson G.V. (2012). Truncated tau and Abeta cooperatively impair mitochondria in primary neurons. Neurobiol. Aging.

[34] Pedros I., Petrov D., Allgaier M., Sureda F., Barroso E., Beas-Zarate C., Auladell C., Pallas M., Vazquez-Carrera M., Casadesus G. (2014). Early alterations in energy metabolism in the hippocampus of APPswe/PS1dE9 mouse model of Alzheimer’s disease. Biochim. Biophys. Acta.

[35] Manczak M., Park B.S., Jung Y., Reddy P.H. (2004). Differential expression of oxidative phosphorylation genes in patients with Alzheimer’s disease: Implications for early mitochondrial dysfunction and oxidative damage. Neuromol. Med..

[36] Leuner K., Hauptmann S., Abdel-Kader R., Scherping I., Keil U., Strosznajder J.B., Eckert A., Muller W.E. (2007). Mitochondrial dysfunction: The first domino in brain aging and Alzheimer’s disease?. Antioxid. Redox Signal..

[37] Hauptmann S., Scherping I., Drose S., Brandt U., Schulz K.L., Jendrach M., Leuner K., Eckert A., Muller W.E. (2009). Mitochondrial dysfunction: An early event in Alzheimer pathology accumulates with age in AD transgenic mice. Neurobiol. Aging.

[38] Chen J.X., Yan S.S. (2010). Role of mitochondrial amyloid-beta in Alzheimer’s disease. J. Alzheimer’s Dis..

[39] Reddy P.H. (2009). Amyloid beta, mitochondrial structural and functional dynamics in Alzheimer’s disease. Exp. Neurol..

[40] Swerdlow R.H. (2012). Mitochondria and cell bioenergetics: Increasingly recognized components and a possible etiologic cause of Alzheimer’s disease. Antioxid. Redox Signal..

[41] Manczak M., Reddy P.H. (2012). Abnormal interaction of VDAC1 with amyloid beta and phosphorylated tau causes mitochondrial dysfunction in Alzheimer’s disease. Hum. Mol. Genet..

[42] Ren R., Zhang Y., Li B., Wu Y., Li B. (2011). Effect of beta-amyloid (25–35) on mitochondrial function and expression of mitochondrial permeability transition pore proteins in rat hippocampal neurons. J. Cell. Biochem..

[43] Cuadrado-Tejedor M., Vilarino M., Cabodevilla F., Del Rio J., Frechilla D., Perez-Mediavilla A. (2011). Enhanced expression of the voltage-dependent anion channel 1 (VDAC1) in Alzheimer’s disease transgenic mice: An insight into the pathogenic effects of amyloid-beta. J. Alzheimer’s Dis..

[44] Sultana R., Poon H.F., Cai J., Pierce W.M., Merchant M., Klein J.B., Markesbery W.R., Butterfield D.A. (2006). Identification of nitrated proteins in Alzheimer’s disease brain using a redox proteomics approach. Neurobiol. Dis..

[45] Smilansky A., Dangoor L., Nakdimon I., Ben-Hail D., Mizrachi D., Shoshan-Barmatz V. (2015). The Voltage-dependent Anion Channel 1 Mediates Amyloid beta Toxicity and Represents a Potential Target for Alzheimer Disease Therapy. J. Biol. Chem..

[46] Thinnes F.P. (2011). Apoptogenic interactions of plasmalemmal type-1 VDAC and Abeta peptides via GxxxG motifs induce Alzheimer’s disease—A basic model of apoptosis?. Wien. Med. Wochenschr..

[47] Jope R.S., Yuskaitis C.J., Beurel E. (2007). Glycogen synthase kinase-3 (GSK3): Inflammation, diseases, and therapeutics. Neurochem. Res..

[48] Pastorino J.G., Hoek J.B., Shulga N. (2005). Activation of glycogen synthase kinase 3beta disrupts the binding of hexokinase II to mitochondria by phosphorylating voltage-dependent anion channel and potentiates chemotherapy-induced cytotoxicity. Cancer Res..

[49] Abu-Hamad S., Arbel N., Calo D., Arzoine L., Israelson A., Keinan N., Ben-Romano R., Friedman O., Shoshan-Barmatz V. (2009). The VDAC1 N-terminus is essential both for apoptosis and the protective effect of anti-apoptotic proteins. J. Cell Sci..

[50] Arzoine L., Zilberberg N., Ben-Romano R., Shoshan-Barmatz V. (2009). Voltage-dependent anion channel 1-based peptides interact with hexokinase to prevent its anti-apoptotic activity. J. Biol. Chem..

[51] Ben-Hail D., Begas-Shvartz R., Shalev M., Shteinfer-Kuzmine A., Gruzman A., Reina S., De Pinto V., Shoshan-Barmatz V. (2016). Novel Compounds Targeting the Mitochondrial Protein VDAC1 Inhibit Apoptosis and Protect against Mitochondrial Dysfunction. J. Biol. Chem..

[52] Zhang E., Mohammed Al-Amily I., Mohammed S., Luan C., Asplund O., Ahmed M., Ye Y., Ben-Hail D., Soni A., Vishnu N. (2019). Preserving Insulin Secretion in Diabetes by Inhibiting VDAC1 Overexpression and Surface Translocation in beta Cells. Cell Metab..

[53] Kim J., Gupta R., Blanco L.P., Yang S., Shteinfer-Kuzmine A., Wang K., Zhu J., Yoon H.E., Wang X., Kerkhofs M. (2019). VDAC oligomers form mitochondrial pores to release mtDNA fragments and promote lupus-like disease. Science.

[54] Verma A., Pittala S., Alhozeel B., Shteinfer-Kuzmine A., Ohana E., Gupta R., Chung J.H., Shoshan-Barmatz V. (2022). The role of the mitochondrial protein VDAC1 in inflammatory bowel disease: A potential therapeutic target. Mol. Ther..

[55] Verma A., Shteinfer-Kuzmine A., Kamenetsky N., Pittala S., Paul A., Nahon Crystal E., Ouro A., Chalifa-Caspi V., Pandey S.K., Monsonego A. (2022). Targeting the overexpressed mitochondrial protein VDAC1 in a mouse model of Alzheimer’s disease protects against mitochondrial dysfunction and mitigates brain pathology. Transl. Neurodegener..

[56] Shteinfer-Kuzmine A., Argueti-Ostrovsky S., Leyton-Jaimes M.F., Anand U., Abu-Hamad S., Zalk R., Shoshan-Barmatz V., Israelson A. (2022). Targeting the Mitochondrial Protein VDAC1 as a Potential Therapeutic Strategy in ALS. Int. J. Mol. Sci..

[57] Yu C.H., Davidson S., Harapas C.R., Hilton J.B., Mlodzianoski M.J., Laohamonthonkul P., Louis C., Low R.R.J., Moecking J., De Nardo D. (2020). TDP-43 Triggers Mitochondrial DNA Release via mPTP to Activate cGAS/STING in ALS. Cell.

[58] Rovini A., Gurnev P.A., Beilina A., Queralt-Martin M., Rosencrans W., Cookson M.R., Bezrukov S.M., Rostovtseva T.K. (2020). Molecular mechanism of olesoxime-mediated neuroprotection through targeting alpha-synuclein interaction with mitochondrial VDAC. Cell. Mol. Life Sci..

[59] Bordet T., Buisson B., Michaud M., Drouot C., Galea P., Delaage P., Akentieva N.P., Evers A.S., Covey D.F., Ostuni M.A. (2007). Identification and characterization of cholest-4-en-3-one, oxime (TRO19622), a novel drug candidate for amyotrophic lateral sclerosis. J. Pharmacol. Exp. Ther..

[60] Kolb P.S., Ayaub E.A., Zhou W., Yum V., Dickhout J.G., Ask K. (2015). The therapeutic effects of 4-phenylbutyric acid in maintaining proteostasis. Int. J. Biochem. Cell Biol..

[61] Tiwari S., Gupta P., Singh A., Chaturvedi S., Wahajuddin M., Mishra A., Singh S. (2022). 4-Phenylbutyrate Mitigates the Motor Impairment and Dopaminergic Neuronal Death During Parkinson’s Disease Pathology via Targeting VDAC1 Mediated Mitochondrial Function and Astrocytes Activation. Neurochem. Res..

[62] Shteinfer-Kuzmine A., Argueti S., Gupta R., Shvil N., Abu-Hamad S., Gropper Y., Hoeber J., Magri A., Messina A., Kozlova E.N. (2019). A VDAC1-Derived N-Terminal Peptide Inhibits Mutant SOD1-VDAC1 Interactions and Toxicity in the SOD1 Model of ALS. Front. Cell. Neurosci..

[63] Magri A., Belfiore R., Reina S., Tomasello M.F., Di Rosa M.C., Guarino F., Leggio L., De Pinto V., Messina A. (2016). Hexokinase I N-terminal based peptide prevents the VDAC1-SOD1 G93A interaction and re-establishes ALS cell viability. Sci. Rep..

[64] Magri A., Risiglione P., Caccamo A., Formicola B., Tomasello M.F., Arrigoni C., Zimbone S., Guarino F., Re F., Messina A. (2021). Small Hexokinase 1 Peptide against Toxic SOD1 G93A Mitochondrial Accumulation in ALS Rescues the ATP-Related Respiration. Biomedicines.

[65] Vijayan M., Reddy P.H. (2022). Reduced VDAC1, Maintained Mitochondrial Dynamics and Enhanced Mitochondrial Biogenesis in a Transgenic Tau Mouse Model of Alzheimer’s Disease. Int. J. Mol. Sci..

[66] Ben-Shlomo Y., Darweesh S., Llibre-Guerra J., Marras C., San Luciano M., Tanner C. (2024). The epidemiology of Parkinson’s disease. Lancet.

[67] De Miranda B.R., Goldman S.M., Miller G.W., Greenamyre J.T., Dorsey E.R. (2022). Preventing Parkinson’s Disease: An Environmental Agenda. J. Parkinson’s Dis..

[68] Bloem B.R., Okun M.S., Klein C. (2021). Parkinson’s disease. Lancet.

[69] Beitz J.M. (2014). Parkinson’s disease: A review. Front. Biosci. (Schol. Ed.).

[70] Thomas B., Beal M.F. (2007). Parkinson’s disease. Hum. Mol. Genet..

[71] Klein C., Westenberger A. (2012). Genetics of Parkinson’s disease. Cold Spring Harb. Perspect. Med..

[72] Lwin A., Orvisky E., Goker-Alpan O., LaMarca M.E., Sidransky E. (2004). Glucocerebrosidase mutations in subjects with parkinsonism. Mol. Genet. Metab..

[73] Billingsley K.J., Bandres-Ciga S., Saez-Atienzar S., Singleton A.B. (2018). Genetic risk factors in Parkinson’s disease. Cell Tissue Res..

[74] Braak H., Del Tredici K., Rub U., de Vos R.A., Jansen Steur E.N., Braak E. (2003). Staging of brain pathology related to sporadic Parkinson’s disease. Neurobiol. Aging.

[75] Spillantini M.G., Schmidt M.L., Lee V.M., Trojanowski J.Q., Jakes R., Goedert M. (1997). Alpha-synuclein in Lewy bodies. Nature.

[76] Yang Y., Shi Y., Schweighauser M., Zhang X., Kotecha A., Murzin A.G., Garringer H.J., Cullinane P.W., Saito Y., Foroud T. (2022). Structures of alpha-synuclein filaments from human brains with Lewy pathology. Nature.

[77] Guo Y., Sun Y., Song Z., Zheng W., Xiong W., Yang Y., Yuan L., Deng H. (2021). Genetic Analysis and Literature Review of SNCA Variants in Parkinson’s Disease. Front. Aging Neurosci..

[78] Morris H.R., Spillantini M.G., Sue C.M., Williams-Gray C.H. (2024). The pathogenesis of Parkinson’s disease. Lancet.

[79] Mani S., Sevanan M., Krishnamoorthy A., Sekar S. (2021). A systematic review of molecular approaches that link mitochondrial dysfunction and neuroinflammation in Parkinson’s disease. Neurol. Sci..

[80] Langston J.W. (2017). The MPTP Story. J. Parkinson’s Dis..

[81] Betarbet R., Sherer T.B., MacKenzie G., Garcia-Osuna M., Panov A.V., Greenamyre J.T. (2000). Chronic systemic pesticide exposure reproduces features of Parkinson’s disease. Nat. Neurosci..

[82] He Y., Wang W., Yang T., Thomas E.R., Dai R., Li X. (2022). The Potential Role of Voltage-Dependent Anion Channel in the Treatment of Parkinson’s Disease. Oxid. Med. Cell. Longev..

[83] Ham S.J., Lee D., Yoo H., Jun K., Shin H., Chung J. (2020). Decision between mitophagy and apoptosis by Parkin via VDAC1 ubiquitination. Proc. Natl. Acad. Sci. USA.

[84] Pickrell A.M., Youle R.J. (2015). The roles of PINK1, parkin, and mitochondrial fidelity in Parkinson’s disease. Neuron.

[85] Dimasuay K.G., Schaunaman N., Martin R.J., Pavelka N., Kolakowski C., Gottlieb R.A., Holguin F., Chu H.W. (2020). Parkin, an E3 ubiquitin ligase, enhances airway mitochondrial DNA release and inflammation. Thorax.

[86] Tomasello F., Messina A., Lartigue L., Schembri L., Medina C., Reina S., Thoraval D., Crouzet M., Ichas F., De Pinto V. (2009). Outer membrane VDAC1 controls permeability transition of the inner mitochondrial membrane in cellulo during stress-induced apoptosis. Cell Res..

[87] Liu Y., Ma X., Fujioka H., Liu J., Chen S., Zhu X. (2019). DJ-1 regulates the integrity and function of ER-mitochondria association through interaction with IP3R3-Grp75-VDAC1. Proc. Natl. Acad. Sci. USA.

[88] Wu H., Carvalho P., Voeltz G.K. (2018). Here, there, and everywhere: The importance of ER membrane contact sites. Science.

[89] Queralt-Martin M., Bergdoll L., Teijido O., Munshi N., Jacobs D., Kuszak A.J., Protchenko O., Reina S., Magri A., De Pinto V. (2020). A lower affinity to cytosolic proteins reveals VDAC3 isoform-specific role in mitochondrial biology. J. Gen. Physiol..

[90] Rostovtseva T.K., Gurnev P.A., Protchenko O., Hoogerheide D.P., Yap T.L., Philpott C.C., Lee J.C., Bezrukov S.M. (2015). alpha-Synuclein Shows High Affinity Interaction with Voltage-dependent Anion Channel, Suggesting Mechanisms of Mitochondrial Regulation and Toxicity in Parkinson Disease. J. Biol. Chem..

[91] Lu L., Zhang C., Cai Q., Lu Q., Duan C., Zhu Y., Yang H. (2013). Voltage-dependent anion channel involved in the alpha-synuclein-induced dopaminergic neuron toxicity in rats. Acta Biochim. Biophys. Sin..

[92] Rosencrans W.M., Aguilella V.M., Rostovtseva T.K., Bezrukov S.M. (2021). alpha-Synuclein emerges as a potent regulator of VDAC-facilitated calcium transport. Cell Calcium.

[93] Chu Y., Goldman J.G., Kelly L., He Y., Waliczek T., Kordower J.H. (2014). Abnormal alpha-synuclein reduces nigral voltage-dependent anion channel 1 in sporadic and experimental Parkinson’s disease. Neurobiol. Dis..

[94] Alberio T., Mammucari C., D’Agostino G., Rizzuto R., Fasano M. (2014). Altered dopamine homeostasis differentially affects mitochondrial voltage-dependent anion channels turnover. Biochim. Biophys. Acta.

[95] Xiong Y., Ding H., Xu M., Gao J. (2009). Protective effects of asiatic acid on rotenone- or H_2_O_2_-induced injury in SH-SY5Y cells. Neurochem. Res..

[96] Periquet M., Corti O., Jacquier S., Brice A. (2005). Proteomic analysis of parkin knockout mice: Alterations in energy metabolism, protein handling and synaptic function. J. Neurochem..

[97] Magalingam K.B., Somanath S.D., Ramdas P., Haleagrahara N., Radhakrishnan A.K. (2022). 6-Hydroxydopamine Induces Neurodegeneration in Terminally Differentiated SH-SY5Y Neuroblastoma Cells via Enrichment of the Nucleosomal Degradation Pathway: A Global Proteomics Approach. J. Mol. Neurosci..

[98] Feng S., Gui J., Qin B., Ye J., Zhao Q., Guo A., Sang M., Sun X. (2024). Resveratrol Inhibits VDAC1-Mediated Mitochondrial Dysfunction to Mitigate Pathological Progression in Parkinson’s Disease Model. Mol. Neurobiol..

[99] Mead R.J., Shan N., Reiser H.J., Marshall F., Shaw P.J. (2023). Amyotrophic lateral sclerosis: A neurodegenerative disorder poised for successful therapeutic translation. Nat. Rev. Drug Discov..

[100] Israelson A., Ditsworth D., Sun S., Song S., Liang J., Hruska-Plochan M., McAlonis-Downes M., Abu-Hamad S., Zoltsman G., Shani T. (2015). Macrophage migration inhibitory factor as a chaperone inhibiting accumulation of misfolded SOD1. Neuron.

[101] Feldman E.L., Goutman S.A., Petri S., Mazzini L., Savelieff M.G., Shaw P.J., Sobue G. (2022). Amyotrophic lateral sclerosis. Lancet.

[102] Wang H., Guan L., Deng M. (2023). Recent progress of the genetics of amyotrophic lateral sclerosis and challenges of gene therapy. Front. Neurosci..

[103] Ling S.C., Polymenidou M., Cleveland D.W. (2013). Converging mechanisms in ALS and FTD: Disrupted RNA and protein homeostasis. Neuron.

[104] Masrori P., Van Damme P. (2020). Amyotrophic lateral sclerosis: A clinical review. Eur. J. Neurol..

[105] Ramesh N., Pandey U.B. (2017). Autophagy Dysregulation in ALS: When Protein Aggregates Get Out of Hand. Front. Mol. Neurosci..

[106] Carri M.T., Valle C., Bozzo F., Cozzolino M. (2015). Oxidative stress and mitochondrial damage: Importance in non-SOD1 ALS. Front. Cell. Neurosci..

[107] Le Gall L., Anakor E., Connolly O., Vijayakumar U.G., Duddy W.J., Duguez S. (2020). Molecular and Cellular Mechanisms Affected in ALS. J. Pers. Med..

[108] King A.E., Woodhouse A., Kirkcaldie M.T., Vickers J.C. (2016). Excitotoxicity in ALS: Overstimulation, or overreaction?. Exp. Neurol..

[109] Philips T., Rothstein J.D. (2014). Glial cells in amyotrophic lateral sclerosis. Exp. Neurol..

[110] Philips T., Robberecht W. (2011). Neuroinflammation in amyotrophic lateral sclerosis: Role of glial activation in motor neuron disease. Lancet Neurol..

[111] Bowerman M., Vincent T., Scamps F., Perrin F.E., Camu W., Raoul C. (2013). Neuroimmunity dynamics and the development of therapeutic strategies for amyotrophic lateral sclerosis. Front. Cell. Neurosci..

[112] Vielhaber S., Winkler K., Kirches E., Kunz D., Buchner M., Feistner H., Elger C.E., Ludolph A.C., Riepe M.W., Kunz W.S. (1999). Visualization of defective mitochondrial function in skeletal muscle fibers of patients with sporadic amyotrophic lateral sclerosis. J. Neurol. Sci..

[113] Ferraiuolo L., Kirby J., Grierson A.J., Sendtner M., Shaw P.J. (2011). Molecular pathways of motor neuron injury in amyotrophic lateral sclerosis. Nat. Rev. Neurol..

[114] Jhanji R., Behl T., Sehgal A., Bungau S. (2021). Mitochondrial dysfunction and traffic jams in amyotrophic lateral sclerosis. Mitochondrion.

[115] Duffy L.M., Chapman A.L., Shaw P.J., Grierson A.J. (2011). Review: The role of mitochondria in the pathogenesis of amyotrophic lateral sclerosis. Neuropathol. Appl. Neurobiol..

[116] Sasaki S., Iwata M. (2007). Mitochondrial alterations in the spinal cord of patients with sporadic amyotrophic lateral sclerosis. J. Neuropathol. Exp. Neurol..

[117] Vande Velde C., McDonald K.K., Boukhedimi Y., McAlonis-Downes M., Lobsiger C.S., Bel Hadj S., Zandona A., Julien J.P., Shah S.B., Cleveland D.W. (2011). Misfolded SOD1 associated with motor neuron mitochondria alters mitochondrial shape and distribution prior to clinical onset. PLoS ONE.

[118] Bilsland L.G., Sahai E., Kelly G., Golding M., Greensmith L., Schiavo G. (2010). Deficits in axonal transport precede ALS symptoms in vivo. Proc. Natl. Acad. Sci. USA.

[119] Zhao J., Wang X., Huo Z., Chen Y., Liu J., Zhao Z., Meng F., Su Q., Bao W., Zhang L. (2022). The Impact of Mitochondrial Dysfunction in Amyotrophic Lateral Sclerosis. Cells.

[120] Debska-Vielhaber G., Miller I., Peeva V., Zuschratter W., Walczak J., Schreiber S., Petri S., Machts J., Vogt S., Szczepanowska J. (2021). Impairment of mitochondrial oxidative phosphorylation in skin fibroblasts of SALS and FALS patients is rescued by in vitro treatment with ROS scavengers. Exp. Neurol..

[121] Lopez-Gonzalez R., Lu Y., Gendron T.F., Karydas A., Tran H., Yang D., Petrucelli L., Miller B.L., Almeida S., Gao F.B. (2016). Poly(GR) in C9ORF72-Related ALS/FTD Compromises Mitochondrial Function and Increases Oxidative Stress and DNA Damage in iPSC-Derived Motor Neurons. Neuron.

[122] Jankovic M., Novakovic I., Gamil Anwar Dawod P., Gamil Anwar Dawod A., Drinic A., Abdel Motaleb F.I., Ducic S., Nikolic D. (2021). Current Concepts on Genetic Aspects of Mitochondrial Dysfunction in Amyotrophic Lateral Sclerosis. Int. J. Mol. Sci..

[123] Israelson A., Arbel N., Da Cruz S., Ilieva H., Yamanaka K., Shoshan-Barmatz V., Cleveland D.W. (2010). Misfolded mutant SOD1 directly inhibits VDAC1 conductance in a mouse model of inherited ALS. Neuron.

[124] Golshani-Hebroni S.G., Bessman S.P. (1997). Hexokinase binding to mitochondria: A basis for proliferative energy metabolism. J. Bioenerg. Biomembr..

[125] Zaid H., Abu-Hamad S., Israelson A., Nathan I., Shoshan-Barmatz V. (2005). The voltage-dependent anion channel-1 modulates apoptotic cell death. Cell Death Differ..

[126] Abu-Hamad S., Zaid H., Israelson A., Nahon E., Shoshan-Barmatz V. (2008). Hexokinase-I protection against apoptotic cell death is mediated via interaction with the voltage-dependent anion channel-1: Mapping the site of binding. J. Biol. Chem..

[127] Azoulay-Zohar H., Israelson A., Abu-Hamad S., Shoshan-Barmatz V. (2004). In self-defence: Hexokinase promotes voltage-dependent anion channel closure and prevents mitochondria-mediated apoptotic cell death. Biochem. J..

[128] Pittala M.G.G., Reina S., Cubisino S.A.M., Cucina A., Formicola B., Cunsolo V., Foti S., Saletti R., Messina A. (2020). Post-Translational Modification Analysis of VDAC1 in ALS-SOD1 Model Cells Reveals Specific Asparagine and Glutamine Deamidation. Antioxidants.

[129] Tan W., Naniche N., Bogush A., Pedrini S., Trotti D., Pasinelli P. (2013). Small peptides against the mutant SOD1/Bcl-2 toxic mitochondrial complex restore mitochondrial function and cell viability in mutant SOD1-mediated ALS. J. Neurosci..

[130] Galluzzi L., Blomgren K., Kroemer G. (2009). Mitochondrial membrane permeabilization in neuronal injury. Nat. Rev. Neurosci..

[131] Pedrini S., Sau D., Guareschi S., Bogush M., Brown R.H., Naniche N., Kia A., Trotti D., Pasinelli P. (2010). ALS-linked mutant SOD1 damages mitochondria by promoting conformational changes in Bcl-2. Hum. Mol. Genet..

[132] Magri A., Lipari C.L.R., Caccamo A., Battiato G., Conti Nibali S., De Pinto V., Guarino F., Messina A. (2024). AAV-mediated upregulation of VDAC1 rescues the mitochondrial respiration and sirtuins expression in a SOD1 mouse model of inherited ALS. Cell Death Discov..

[133] Sunyach C., Michaud M., Arnoux T., Bernard-Marissal N., Aebischer J., Latyszenok V., Gouarne C., Raoul C., Pruss R.M., Bordet T. (2012). Olesoxime delays muscle denervation, astrogliosis, microglial activation and motoneuron death in an ALS mouse model. Neuropharmacology.

[134] Lenglet T., Lacomblez L., Abitbol J.L., Ludolph A., Mora J.S., Robberecht W., Shaw P.J., Pruss R.M., Cuvier V., Meininger V. (2014). A phase II-III trial of olesoxime in subjects with amyotrophic lateral sclerosis. Eur. J. Neurol..

[135] Davis S.A., Itaman S., Khalid-Janney C.M., Sherard J.A., Dowell J.A., Cairns N.J., Gitcho M.A. (2018). TDP-43 interacts with mitochondrial proteins critical for mitophagy and mitochondrial dynamics. Neurosci. Lett..

[136] Szabadkai G., Bianchi K., Varnai P., De Stefani D., Wieckowski M.R., Cavagna D., Nagy A.I., Balla T., Rizzuto R. (2006). Chaperone-mediated coupling of endoplasmic reticulum and mitochondrial Ca^2+^ channels. J. Cell Biol..

[137] Pilotto F., Schmitz A., Maharjan N., Diab R., Odriozola A., Tripathi P., Yamoah A., Scheidegger O., Oestmann A., Dennys C.N. (2022). PolyGA targets the ER stress-adaptive response by impairing GRP75 function at the MAM in C9ORF72-ALS/FTD. Acta Neuropathol..

[138] Alvarez-Mora M.I., Garrabou G., Barcos T., Garcia-Garcia F., Grillo-Risco R., Peruga E., Gort L., Borrego-Ecija S., Sanchez-Valle R., Canto-Santos J. (2022). Bioenergetic and Autophagic Characterization of Skin Fibroblasts from C9orf72 Patients. Antioxidants.

[139] Tabrizi S.J., Flower M.D., Ross C.A., Wild E.J. (2020). Huntington disease: New insights into molecular pathogenesis and therapeutic opportunities. Nat. Rev. Neurol..

[140] Jurcau A., Jurcau C.M. (2023). Mitochondria in Huntington’s disease: Implications in pathogenesis and mitochondrial-targeted therapeutic strategies. Neural Regen. Res..

[141] Karachitos A., Grobys D., Kulczynska K., Sobusiak A., Kmita H. (2016). The Association of VDAC with Cell Viability of PC12 Model of Huntington’s Disease. Front. Oncol..

[142] Brondani M., Roginski A.C., Ribeiro R.T., de Medeiros M.P., Hoffmann C.I.H., Wajner M., Leipnitz G., Seminotti B. (2023). Mitochondrial dysfunction, oxidative stress, ER stress and mitochondria-ER crosstalk alterations in a chemical rat model of Huntington’s disease: Potential benefits of bezafibrate. Toxicol. Lett..

[143] El-Emam M.A., Sheta E., El-Abhar H.S., Abdallah D.M., El Kerdawy A.M., Eldehna W.M., Gowayed M.A. (2024). Morin suppresses mTORc1/IRE-1alpha/JNK and IP3R-VDAC-1 pathways: Crucial mechanisms in apoptosis and mitophagy inhibition in experimental Huntington’s disease, supported by in silico molecular docking simulations. Life Sci..

[144] Bordet T., Berna P., Abitbol J.L., Pruss R.M. (2010). Olesoxime (TRO19622): A Novel Mitochondrial-Targeted Neuroprotective Compound. Pharmaceuticals.

[145] Bertini E., Dessaud E., Mercuri E., Muntoni F., Kirschner J., Reid C., Lusakowska A., Comi G.P., Cuisset J.M., Abitbol J.L. (2017). Safety and efficacy of olesoxime in patients with type 2 or non-ambulatory type 3 spinal muscular atrophy: A randomised, double-blind, placebo-controlled phase 2 trial. Lancet Neurol..

[146] Muntoni F., Bertini E., Comi G., Kirschner J., Lusakowska A., Mercuri E., Scoto M., van der Pol W.L., Vuillerot C., Burdeska A. (2020). Long-term follow-up of patients with type 2 and non-ambulant type 3 spinal muscular atrophy (SMA) treated with olesoxime in the OLEOS trial. Neuromuscul. Disord..

[147] Mishra E., Thakur M.K. (2022). Alterations in hippocampal mitochondrial dynamics are associated with neurodegeneration and recognition memory decline in old male mice. Biogerontology.

[148] Kielar C., Wishart T.M., Palmer A., Dihanich S., Wong A.M., Macauley S.L., Chan C.H., Sands M.S., Pearce D.A., Cooper J.D. (2009). Molecular correlates of axonal and synaptic pathology in mouse models of Batten disease. Hum. Mol. Genet..

